# Quality Improvement Intervention Decreases Pain and Adverse Events Due to Heel Lances in Infants

**DOI:** 10.3390/children11121456

**Published:** 2024-11-28

**Authors:** Betty Noergaard, Helle Brems Olesen, Ulla List Toennesen, Jesper Fenger-Gron, Poul-Erik Kofoed

**Affiliations:** 1 Department of Paediatrics and Adolescent Medicine, Lillebaelt Hospital, University Hospital of Southern Denmark, Sygehusvej 24, 6000 Kolding, Denmark; helle.brems.olesen@rsyd.dk (H.B.O.); jesper.fenger-groen@rsyd.dk (J.F.-G.); poul.erik.kofoed@rsyd.dk (P.-E.K.); 2 Department of Clinical Biochemistry and Immunology, Lillebaelt Hospital, University Hospital of Southern Denmark, Sygehusvej 24, 6000 Kolding, Denmark; ulla.list.toennesen@rsyd.dk; 3 Institute of Regional Health Research, Faculty of Health Sciences, University of Southern Denmark, Campusvej 55, 5230 Odense, Denmark

**Keywords:** infant, newborn, pain, heel, parent, patient safety, risk management, intensive care units, neonatal

## Abstract

Background: Studies have investigated ways to reduce infants’ pain during heel lancing, but research on preventing adverse events is scarce. This study investigated whether or not the number of infants with normal comfort (>8 and ≤14), distress (≤4), and pain (≤4) scores increased and whether or not the number of adverse events (blue and/or edematous heels and improperly placed incisions) decreased during and after heel lancing following an intervention. Methods: A pre- and post-quality improvement intervention including 189 and 186 heel lances, respectively, in infants (postmenstrual age ≥ 28 + 0 to ≤ 43 + 6 weeks) was conducted in May to July 2020 and April to July 2022. The intervention comprised five initiatives: skin-to-skin contact, comforting, sucrose/breastfeeding, warming cold heels, and ergonomics for staff. ComfortNeo score, along with distress and pain scores assessed the infants’ pain and discomfort before, during, and after heel lancing. Adverse events were assessed visually. Results: Post-intervention, there was a significant increase in the number of infants with normal pain and distress scores during (86% to 95%, *p* = 0.01, and 82% to 93%, *p* = 0.01, respectively) and after (95% to 99%, *p* = 0.04, and 90% to 99%, *p* < 0.01, respectively) heel lancing compared to the pre-intervention. A non-significant reduction in the number of adverse events was observed (41 to 30, *p* = 0.17). Conclusions: Cost-free procedural changes can decrease pain, discomfort, and adverse events in infants undergoing heel lancing. Painful procedures should be evaluated and optimized. Staff and parents should collaborate to manage pain and adverse events.

## 1. Introduction

During hospitalization in a neonatal intensive care unit (NICU), the treatment and care of infants involve various clinical and diagnostic procedures that expose them to pain and stress. A study reported that, on average, each infant was exposed to 13.9 painful procedures daily, with capillary blood sampling (heel lance) being one of the most frequent [1]. Additionally, premature infants are more vulnerable and exhibit higher sensitivity to pain compared to full-term infants [2].

These repeated painful procedures can lead to long-term neurodevelopmental disabilities, which have major implications for both cognitive and emotional development [3]. Further knowledge is required that involves effective pain management that reduces pain in neonatal infants.

Studies on pain reduction and infants’ comfort during heel lancing recommend warming infants’ heels before the procedure, providing sucrose, breastfeeding, skin-to-skin contact (SSC), and the involvement of parents actively comforting their infants during the procedure [4,5,6,7,8,9]. However, studies on how to prevent adverse events are scarce or lacking.

During our routine biannual quality monitoring of infant comfort during heel lancing, we found that our practice did not fully meet the aforementioned recommendations. In addition, the staff reported adverse events, such as blue and/or edematous heels, severe bruising and burns following heel warming, and improperly placed incisions (unpublished data). These findings raised concerns and were identified as quality issues because of the potential adverse effects on these vulnerable infants.

Through quality monitoring, it became evident that, although heel lances are frequent and seemingly simple procedures, they turned out to be complex and could be influenced by various factors, including gestational age, weight, positioning during the procedure, heel warming, the presence of parents, non-pharmacological nursing comfort measures, and the ergonomics of biomedical laboratory scientists (BLSs).

**Figure 1:**
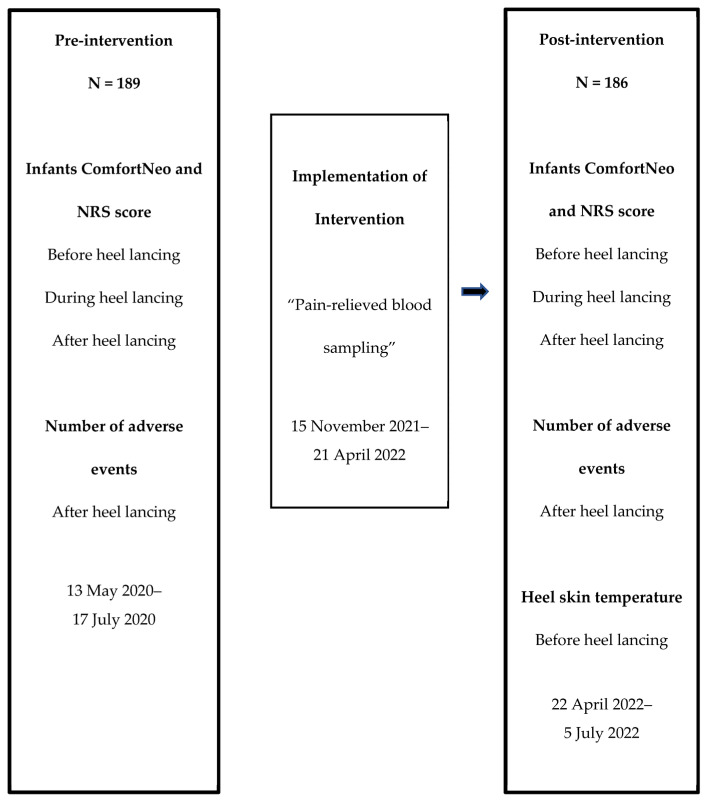
Timeframe of the study.

Therefore, we decided to plan and implement a pre- and post-quality improvement intervention study called ‘Pain-relieved blood sampling’ (Figure 1) with the aim of improving infants’ comfort and reducing the number of adverse events related to heel lances.

In the pre-intervention (baseline) phase, we found that all infants’ heels were warmed with a glove filled with hot tap water before heel lancing and that the infants were comforted (see methods section), either by their parents or by nurses. Furthermore, all infants received sucrose and/or breastfeeding. Forty percent of infants had SSC with their parents during the procedure. However, we found that infants having SSC experienced only a minor increase in the mean ComfortNeo score (from 11.3 to 14.2) from before to during the heel lance procedure compared to infants who did not receive SSC (from 11.5 to 14.3). This suggests that the pain relief provided by SSC was minimal and that the infants’ pain may have been partially managed through other measures, such as heel warming, sucrose or breastfeeding, and comforting from parents or nurses [10]. In a randomized controlled trial (RCT) conducted before the implementation of the intervention (Pain-relieved blood sampling), no differences were found between the warming methods routinely used in Denmark (glove, gel pack, and blanket) regarding infant comfort and blood sample quality [11]. However, when the BLS indicated a poor grip on the foot during sampling, the infants’ discomfort, degree of hemolysis, and blood sampling time increased (unpublished data).

Furthermore, the RCT found that the number of adverse events increased significantly, with most injuries, such as bruises or swelling of the heel, occurring in heels with a skin temperature above 37.0 °C before the heel lance. Additionally, most injuries (non-significant) were seen after warming with gloves containing hot water [11].

The aim of the current study was to answer the following question: will the number of infants having a normal comfort, distress, and pain score increase (see methods section), and will the number of adverse events (blue heels edematous heels and improperly placed incisions) decrease during and after heel lancing following the implementation of the ‘Pain-relieved blood sampling’ intervention?

## 2. Materials and Methods

### 2.1. Design and Setting

This quality improvement study, designed as a pre- and post-intervention study to evaluate the intervention ‘Pain-relieved blood sampling’, was conducted from 13 May 2020, to 17 July 2020, and from 22 April 2022, to 5 July 2022 (Figure 1).

The study was conducted in collaboration with the Department of Clinical Biochemistry and Immunology and the Department of Pediatrics and Adolescent Medicine at Lillebaelt Hospital, University Hospital of Southern Denmark, Kolding. It took place at a 19-bed level II NICU, which annually treats approximately 700 sick newborns with a gestational age of ≥28 weeks. Except for those with infectious diseases, parents and siblings had unlimited access to the unit. Armchairs or hospital beds were provided next to the infants’ incubators or cradles, and parents were allowed to sleep next to their infants or in a patient hotel adjacent to the NICU.

### 2.2. Participants

Recruitment was based on heel lances, and infants could participate more than once during hospitalization in the NICU.

Eligibility for inclusion included heel lances performed by BLSs in infants with a postmenstrual age of ≥28 + 0 weeks and ≤43 + 6 weeks. Heel lances were excluded if (1) they were performed in infants less than 2 h after delivery or (2) the infant’s heel on which the procedure was performed was bruised before blood sampling.

BLSs are trained and experienced in performing capillary and venous blood sampling in infants. Like the nurses, BLSs are also authorized to administer sucrose, recommend breastfeeding, and suggest parents engage in SSC. In infant blood sampling situations, the BLS and nurse collaborate to administer sucrose, SSC, or other procedures to ensure pain relief.

### 2.3. Instruments

The nurses recorded weight, postmenstrual age, position during the procedure, and pain-relieving actions in the infants undergoing heel lancing. Using the pain assessment tool ComfortNeo and numeric rating scores (NRS) for pain and distress [12,13,14], nurses certified in ComfortNeo measured the infants’ comfort, pain, and distress levels at three time points relative to each heel lance: (1) approximately 5 min before (before), (2) during (during), and (3) 5 min after (after).

The ComfortNeo score consists of six behavioral dimensions: alertness, calmness/agitation, crying (spontaneously breathing infants only), respiratory response (invasively ventilated infants only), facial tension, muscle tone, and body movements. The infants are observed for 2 min to rate each dimension on a scale of 1 to 5, with higher scores indicating a greater response. The total score ranges from 6 to 30. A ComfortNeo score of ≥14 indicates that the infant is in pain or discomfort. A score of ≤8 suggests that the infant may be under the influence of opioid or sedative medication or has become flaccid due to prematurity or illness. A normal ComfortNeo score is >8 and ≤14 [14]. NRS scores range from 0 to 10, with higher values indicating greater pain and distress levels. A score of >4 suggests that the infant is in pain or is distressed. A normal distress and pain score is ≤4 [14].

Data were recorded on a registration form developed for this study. Two hours after the procedure, a nurse visually examined the heels for any adverse events, such as blue and/or edema heels, or improperly placed incisions.

After each heel lance, the BLS recorded the number of incisions needed to obtain the blood sample and rated the amount of heel squeezing required on a 5-point Likert scale, with scores ranging from 0 (no squeezing) to 5 (more squeezing). In addition, they also assessed their grip on the foot and their ergonomic position during the heel lancing, using a 5-point Likert scale, with scores ranging from 0 (poor) to 5 (good).

Furthermore, the BLS stated their experience (years) performing heel lances on infants.

Finally, in the post-intervention study, nurses recorded the infants’ heel-skin temperatures before the procedure.

### 2.4. Intervention

Results from the pre-intervention study [10] and the RCT [11] were pivotal to designing the intervention, which consisted of five specific initiatives and was implemented as a package named ‘Pain-relieved blood sampling’.

Although the pre-intervention study found that SSC had no or only a minor additional pain-relieving effect on infants’ comfort [10], we included the initiative *‘Infants were placed skin-to-skin (if possible)*’ in the intervention because other studies have shown that SSC not only has a pain-relieving effect [15,16] but also a valuable effect on the parenting role [5,8,9].

The initiatives *‘Infants are soothed with sucrose or lactation during the procedure’ and ‘Infants are comforted by their parents and/or a nurse’* have both been recommended previously [5,6,7,9,15]. Comforting is the act of parents or nurses providing physical and emotional support to the infant through actions such as positioning, gentle touch, and soothing speech. It also involves assessing the environment to create a calming and secure atmosphere. Although these initiatives were already part of the department’s guidelines, we included them to ensure that clinical instructions were followed.

The results of the RCT suggested that adverse events were caused by excessively warm heels [11]. Therefore, we decided to evaluate this by including the initiative *‘Infants’ heels are only warmed for 5 min with a cotton blanket from an incubator set at 39.0 °C if heel-skin temperature is <37.0 °C (measured before the procedure)’* in the intervention.

The final initiative, *‘Nurses help BLSs to have the best possible ergonomics’* was implemented because BLS ergonomics had been found to affect infants’ comfort and blood sampling quality (unpublished data). Good ergonomics when performing heel lances is when the BLS experiences having the best possible position to achieve a firm grip on the heel without squeezing, while also maintaining a good ergonomic posture to avoid twisting the back or standing bent forward.

Additionally, simulation training was conducted for BLSs. A nurse trained as a simulation-instructor and a BLS-instructor conducted two-hour lectures with 39 BLSs and 14 nurses. The instructors focused on how BLSs could improve their ergonomics and how collaboration between BLSs, nurses, and parents could enhance the heel lance procedure. Additionally, BLSs gained valuable knowledge regarding pain management.

### 2.5. Outcomes

The primary outcomes were differences in the number of infants with normal comfort, distress, and pain scores during and after heel lance between the pre-intervention and post-intervention groups.

The secondary outcome was the difference in the number of adverse events after heel lance between the pre-intervention and post-intervention groups.

### 2.6. Ethics

The Regional Committee on Health Research Ethics for Southern Denmark indicated that ethical approval was not necessary. The study was conducted in accordance with the Declaration of Helsinki and the protocol was approved by the executive board of Lillebaelt Hospital (journal number 20/53345). Parents were verbally informed about the study. Informed consent for participation was not required as per local legislation (journal number 20/1316).

### 2.7. Statistical Analyses

For group comparisons, the Mann–Whitney U test was used for non-normally distributed data, and the Chi-squared test was used for binary outcomes. Predictors of comfort, distress, pain, and adverse events were analyzed using multiple regression models, with adverse events and comfort, distress, and pain as dependent variables and weight, position, number of incisions, warming, BLS ergonomics, heel squeezing, grip on the foot, BLS experience, and study group as independent variables. Statistical analyses were performed using STATA version 16 (StataCorp, College Station, TX, USA).

An electronic data capture platform (REDCap version 13.7.18) [17,18] was used to record all survey data.

In the evaluation of the intervention’s impact on the number of adverse events, all heel lances were included, whereas only heel lances in infants with a normal comfort, distress, and pain score (ComfortNeo score >8 and ≤14, NRS distress score ≤ 4, and NRS pain score ≤ 4) before the procedure were included in the assessment of the infants’ comfort to avoid results being influenced by other pain-producing causes or infants being flaccid.

We created the variables for BLS ergonomics, grip on the foot, and squeezing by summing the scores within two ranges: 0–2 and 3–5 for each variable. Each variable was then dichotomized as follows: poor and good ergonomics (0–2 and 3–5, respectively), bad and good grip (0–2 and 3–5, respectively), and less and more squeezing (0–2 and 3–5, respectively).

## 3. Results

### 3.1. Participants and Intervention

The pre- and post-intervention groups consisted of 189 heel lances in 81 infants and 186 heel lances in 81 infants, respectively. Infants’ post menstrual age at procedure ranged from 29–46 and 30–42 weeks, respectively, with a mean of 37 weeks in both groups. Infants’ mean weight (last measured) at the procedure was 2820 g and 2919 g, respectively, with a range from 823–5330 g and 995–4360 g, respectively. The pre- and post-intervention groups were similar.

In the post-intervention group, there was a significant change in infants’ position during heel lance procedures. Fewer heel lances were performed on infants placed in incubators (15/182 vs. 64/187), and more heel lances were performed on infants placed in a cradle (78/182 vs. 47/187) or had SSC (89/182 vs. 76/187) than in the pre-intervention group. Furthermore, significantly fewer heel lances were performed on warmed heels (85/184 vs. 186/187, *p* < 0.01) (Table 1).

Comfort data from the 299 heel lances in infants with an initially normal comfort, distress, and pain score (ComfortNeo score >8 and ≤14, NRS distress and NRS pain scores ≤ 4) were analyzed: 152 and 147 in the pre- and post-intervention groups, respectively.

No significant differences in BLS seniority were observed between the pre- and post-intervention groups. In the pre-intervention group, 70% had a seniority of ≤10 years and 30% of ≥11 years, compared to 73% and 27%, respectively, in the post-intervention group.

### 3.2. Infants’ Comfort

When comparing the pre-intervention group with the post-intervention group, no differences were found in the number of infants with a normal ComfortNeo score either before, during, or after heel lancing.

However, in the post-intervention group a significant increase was observed in the number of infants with normal pain and distress scores during (from 86% to 95%, *p* = 0.01 and 82% to 93%, *p* = 0.01, respectively) and after (95% to 99%, *p* = 0.04 and 90% to 99%, *p* < 0.01, respectively) heel lancing compared to the pre-intervention group (Table 2).

From before to during the heel lance procedure, increases in the means of all pain and discomfort scores were seen in both the pre- and post-intervention groups. The ComfortNeo, NRS distress, and NRS pain scores increased by 3.3, 1.9, and 1.5 points and 3.5, 1.6, and 1.3 points, respectively. Likewise, from during to after the heel lance procedure, decreases in the means of the scores were found in both groups (3.2, 1.3, and 1.1 points and 3.7, 1.5, and 1.2 points, respectively).

Multiple regression analyses revealed that, of the included independent variables (weight, position, number of incisions, warming, BLS ergonomics, heel squeezing, grip on the foot, BLS experience, and study group), the number of incisions showed a significant association with infants’ ComfortNeo score (*p* = 0.01), NRS distress score (*p* = 0.01), and NRS pain score (*p* = 0.01) during the heel lancing and with the ComfortNeo score (*p* = 0.02) and NRS distress score (*p* = 0.01) after the heel lancing. Furthermore, the weight of the infants was significantly associated with the NRS pain score (*p* = 0.01) during heel lancing, with low-weight infants experiencing more pain.

Infants in the post-intervention group compared to the pre-intervention group had significantly lower mean NRS pain scores during and after (1.21 vs. 1.61, *p* = 0.05 and 0.06 vs. 0.57, *p* = 0.01, respectively), and lower mean ComfortNeo and NRS distress scores after the heel lance procedure (11.04 vs. 10.45, *p* = 0.05 and 0.19 vs. 0.82, *p* = 0.01, respectively) when they had SSC with a parent. Furthermore, infants placed in a cradle had higher mean NRS pain scores during heel lancing (1.28 vs. 1.12, *p* = 0.03), whereas no other differences related to position were found between the two groups.

In the pre-intervention group, infants placed in skin-to-skin contact (SSC) with a parent exhibited significantly higher mean NRS pain and NRS distress scores following heel lancing compared to infants lying in cradles or incubators (pain: 0.82 vs. 0.17, *p* = 0.01; distress: 0.58 vs. 0.50, *p* = 0.01). However, in the post-intervention group, there were no significant differences in comfort, pain, or distress scores regardless of whether the infants had SSC or not. In the post-intervention group, there were no differences in the infants’ ComfortNeo, NRS distress, or NRS pain scores either during or after the heel lancing when comparing heels that were warmed versus those that were not warmed before the procedure.

### 3.3. Adverse Events

There was a reduction in the number of adverse events (blue heels and/or edematous heels and improperly placed incisions) from 41/189 (22%) to 30/186 (16%) post-intervention (*p* = 0.17).

Multiple regression analysis showed that, of the included independent variables (weight, position, number of incisions, warming, BLS ergonomics, heel squeezing, grip on the foot, BLS experience, and study group), only weight showed a significant (*p* < 0.01) association with adverse events after heel lancing; the smaller the infant, the higher the risk.

The number of adverse events in infants weighing ≤ 2000 g fell from 21/41 (51%) to 6/30 (20%) following the implementation of the intervention (*p* = 0.16).

In both the pre- and post-intervention groups, more adverse events were seen in infants placed in incubators (28% and 46%, respectively) than in infants placed in cradles (19% and 14%, respectively) or with SSC (18% and 13%, respectively) (*p* = 0.33 and *p* < 0.01, respectively).

In the post-intervention group, no difference was observed in the number of adverse events in heels warmed compared to heels not warmed before the heel lance.

## 4. Discussion

To reduce the discomfort, distress, and pain caused by heel lances in infants, a quality improvement intervention called ‘Pain-relieved blood sampling’ was implemented in our NICU.

Several actions have been found to relieve infants’ discomfort and pain during heel lancing [8,9,11,15]. Additionally, combining these interventions has a supplemental effect on infants’ comfort [15,19]. Following these recommendations, the ‘Pain-relieved blood sampling’ intervention consisted of a combination of five initiatives.

Even though the staff already had a strong focus on the recommended non-pharmacological actions before the intervention (40% of the infants were placed in SSC with a parent; most infants were soothed with sucrose and comforted by their parents and/or a nurse), we found that the intervention had a positive impact on the infants’ comfort. This was shown by the substantial increase in the number of infants with normal pain and distress scores during and after heel lancing.

In both the pre- and post-intervention groups few infants were breastfed, although studies indicate that mothers generally wished to breastfeed their infants during the procedures. The recommendation is that, when possible and when mothers are available, infants should be breastfed during the procedures, with consideration given to cultural and social differences. [8,9,15].

Furthermore, in the pre-intervention group, infants with SSC received only slightly better comfort when they received other pain-relieving actions simultaneously [10]. In the post-intervention group, we found that infants with SSC had lower pain and distress scores after heel lancing, supporting the idea that SSC is effective as a non-pharmacological pain-relieving action. One study demonstrated that kangaroo care and oral sucrose were equally effective during repeated painful procedures; however, combining SSC and sucrose did not have an additive beneficial effect [20]. These results are strengthened by studies showing that through involvement and SSC, both mothers and fathers could help infants cope with painful procedures, while their own parental role was strengthened [9,15].

In this study, we encouraged and verbally informed parents about the importance of breastfeeding and SSC for the infants’ comfort during heel lance procedures. Although parents had unrestricted access to the unit and were permitted to sleep next to their infants or in a patient hotel adjacent to the NICU, there is still room for improvement in promoting SSC and breastfeeding. A review investigated the effectiveness of various methods, such as video, written pamphlets, PowerPoint presentations, and on-screen materials, used to inform parents in hospitals about pain management. The review concluded that it is important to explore which methods are most effective in changing parents’ behavior [21]. In our study, however, it was not monitored whether or not parents were actually provided with the intended information or given the opportunity to engage with it, which may have influenced the outcome of the intervention and, consequently, the results.

No differences were found in infant comfort, distress, or pain during heel lance procedures in warmed heels versus heels not warmed, either during or after the procedure. These results corroborate a study that found warming the heels before the procedure distressed the infants but did not have a pain-relieving effect [19]. However, studies performed on stable, healthy term newborns have found significant reductions in infants’ pain and discomfort when the heels were warmed compared to that during routine procedures (unfortunately not described) [4,22] and that warming increased the blood circulation in the heel area and decreased the duration of crying [23].

In previous studies on painful procedures in infants, very little attention has been paid to staff ergonomics. The improved BLS ergonomics and teamwork among BLSs, parents, and nurses could be a possible explanation for the increase in the number of infants with normal pain and distress scores during and after heel lancing in the post-intervention group. Furthermore, a decrease in infant pain was observed when they rested in cradles. Similarly, we found that, in the pre-intervention group, infants had a greater discomfort after heel lancing when had SSC compared to when they were lying in cradles and incubators. However, no such difference was found in the post-intervention group. Before the intervention, BLSs experienced poor working postures while performing heel lancing on infants during SSC. Through the simulation training, BLSs gained tools and techniques to achieve effective ergonomics when the infants were in SSC.

Nonetheless, we found that neither the BLS ergonomics nor grip or work experience had any effect on infants’ comfort, pain, and distress during and after the procedure.

Warming the heels before heel incision increases vasodilation and blood flow in the area, which theoretically helps with blood sampling [19]. However, increased vasodilation could also increase the number of adverse events, especially in premature and low-weight infants because they are more vulnerable. To our knowledge, this study is the first to investigate the association between the warming the heels of infants before heel lancing and the number of adverse events. After the intervention, a considerable, though non-significant, decrease in adverse events following heel lancing was found, with the largest decrease in infants weighing ≤ 2000 g and infants resting in incubators, probably due to their greater vulnerability. Additionally, the study found a correlation between lower birth weight and higher pain scores, consistent with other research indicating that low-birth-weight infants exhibit stronger reactions to painful procedures [2].

### Strengths and Limitations

One strength of the current study is that it was conducted in real-life practice to evaluate quality improvement interventions. Collaboration between the BLSs who performed the heel lances and the staff at our NICU strengthened the study, as it is important to include all key stakeholders in quality improvement studies [24].

In the pre-intervention group, all heels were warmed prior to heel lances without measuring the heel skin temperature, and this may be considered a limitation that we could not compare the results of adverse events and skin temperature between the pre- and post-intervention groups. The results from the RCT [11] were not available before the start of the pre-intervention data collection, and no previous studies have identified heel-skin temperature as a factor when evaluating adverse events.

The ComfortNeo score has been validated to measure prolonged pain in infants admitted to NICUs [12,14] but not acute pain related to procedures, as in this study, which could have affected the results. Using the NRS tools, we were able to assess the infants’ pain and distress in addition to their comfort. According to the instructions, the infants’ ComfortNeo scores should be measured while they are undisturbed. However, in this study, one of the three measurements was performed during the heel lance procedures, whereas before and after the measurements the infants were left undisturbed [12,14]. The ComfortNeo score was chosen because it is recommended as a Danish pain assessment tool for neonates [13], and the nurses at the NICU were certified and familiar with the tool.

Another limitation was the study not including the first heel lance carried out shortly after admission to the NICU. In these acute and stressful situations, it was not considered ethically correct to include heel lance for scientific purposes, making delay of treatment initiation an imminent risk. Moreover, the small sample size meant that we did not have sufficient power to verify the reduction in the number of adverse events in the smallest and most vulnerable infants.

Nevertheless, we included all eligible heel lances performed during the study period, which strengthens the study, as heel lances represent a diversity of infants admitted to the NICU.

Lastly, the study was conducted over a two-year period, during which other changes could have influenced the results [25]. However, apart from the intended changes in the ‘Pain-relieved blood sampling’ intervention, no other changes regarding heel lance procedures were observed.

## 5. Conclusions

The multifaceted intervention ‘Pain-relieved blood sampling’ implemented in a level II NICU subsequently increased the number of infants with a normal pain and distress score during and following heel lancing. The initiative of not warming the infants’ heels when the heel-skin temperature was >37.0 °C decreased the number of adverse events due to heel lances. Infants weighing ≤ 2000 g and infants placed in incubators appeared to have the highest reduction in adverse events, probably due to their greater vulnerability.

Although the results of this study are promising, we need to continuously monitor infants’ comfort, distress, and pain and the number of adverse events to ensure that the clinical instructions are followed, the quality is maintained, and improvements are identified.

Further research is needed to investigate non-pharmacological pain-relieving actions, including warming or non-warming of heels before heel lances, to improve infant comfort and prevent adverse events.

## Figures and Tables

**Table 1:** Pain-relieving actions in relation to heel lances in the pre- and post-intervention groups, respectively.

		Pre-Intervention Group *	Post-Intervention Group **	Pre- vs. Post- Intervention Group *p*-Value
Heel lances/N		189	186	
Position of infant/N (%)				<0.01
	Skin-to-skin	76 (40)	89 (48)	
	Cradle	47 (25)	78 (43)	
	Incubator	64 (34)	15 (8)	
Warming before heel lance/N (%)		186 (99)	85 (46)	<0.01
Comforting during heel lancing ***/N (%)				
	By parents	124 (67)	135 (74)	0.18
	By nurses	89 (48)	75 (41)	0.16
Other pain-relieving actions/N (%)				
	Sucrose	173 (96)	168 (92)	0.14
	Breast milk	16 (8)	19 (10)	0.53
Number of incisions/median (min-max)		1 (1–4)	1 (1–5)	0.51
BLS ergonomics/N (%)				0.70
	Good	160 (88)	161 (87)	
	Poor	22 (12)	25 (13)	
BLS Squeezing/N (%)				0.80
	Less squeezing	122 (67)	127 (68)	
	More squeezing	60 (33)	59 (32)	
BLS Grip on the foot/N (%)				0.76
	Good	167 (92)	169 (91)	
	Bad	15 (8)	17 (9)	

* = position, warming, support, pain relieving, ergonomics, squeezing, grip, and number of incisions are missing for two, two, five, eight, seven, seven, seven, and 10 heel lances, respectively. ** = position, warming, support, pain relieving and number of incisions is missing for four, two, three, three, and four heel lances, respectively. *** = the infant could be comforted by both the parents and a nurse.

**Table 2:** Infants’ ComfortNeo, NRS distress, and NRS pain scores before, during, and after heel lancing in the pre- and post-intervention groups, respectively—data from heel lances in infants who had no pain or discomfort prior to the procedure.

	Heel Lancing	Pre- Intervention Group N = 152	Post- Intervention Group * N = 147	Pre- vs. Post-Intervention Groups *p*-Value
ComfortNeo score Total number of too low/normal/too high [min-max score]	Before	0/152/0 [9—13]	0/147/0 [9—13]	-
	During	4/65/83 [6—26]	1/60/86 [8—25]	0.73
	After	7/131/14 [6—20]	1/132/10 [8—17]	0.09
NRS distress ** score Total number of normal/too high [min-max score]	Before	152/0 [0—4]	147/0 [0—3]	-
	During	124/28 [0—8]	136/11 [0—8]	0.01
	After	137/15 [0—6]	141/2 [0—10]	<0.01
NRS pain *** score Total number of normal/too high [min-max score]	Before	152/0 [0—1]	147/0 [0—4]	-
	During	130/22 [0—8]	139/8 [0—7]	0.01
	After	145/7 [0—6]	142/1 [0—6]	0.04

* = ComfortNeo, NRS distress, and NRS pain scores after heel lances are missing for four heel lances. ** Numeric rating scores for distress. *** Numeric rating scores for pain.

## Data Availability

Data is available for privacy reasons, but can be requested on request.
